# Arrhythmia-related Hospitalization and Comorbid Cannabis Use Disorder: Trend Analysis in US Hospitals (2010-2014)

**DOI:** 10.7759/cureus.5607

**Published:** 2019-09-09

**Authors:** Paul Rahul Jaladi, Viralkumar Patel, Shanthini Kuduva Rajan, Wahida Rashid, Sowmya Madireddy, Temitope Ajibawo, Sundus Imran, Rikinkumar S Patel

**Affiliations:** 1 Internal Medicine, Rajiv Gandhi Institute of Medical Sciences, Kadapa, IND; 2 Internal Medicine, Blake Medical Center, Bradenton, USA; 3 Internal Medicine, Tirunelveli Medical College, Tirunelveli, IND; 4 Internal Medicine, Dhaka Medical College, Dhaka, BGD; 5 Internal Medicine, Mamata Medical College, Khammam, IND; 6 Internal Medicine, Brookdale University Hospital and Medical Center, New York, USA; 7 Neurology, Indiana University School of Medicine, Indianapolis, USA; 8 Psychiatry, Griffin Memorial Hospital, Norman, USA

**Keywords:** national trends, cannabis, marijuana, recreational marijuana, hospitalization, epidemiological studies, arrhythmia, cardiac arrhythmias

## Abstract

Objective

To study the trends of arrhythmia hospitalizations with cannabis use disorders (CUDs) in terms of demographic characteristics and inpatient outcomes.

Methods

We used the nationwide inpatient sample (NIS) data during the post-legalization period (2010-2014) and included 570,556 arrhythmia inpatients (age, 15-54 years), and 14,426 inpatients had comorbid CUD (2.53%). We used the linear-by-linear association test and independent-sample T-test for assessing the change in hospital outcomes in inpatients with CUD.

Results

Arrhythmia hospitalizations with CUD increased by 31% (2010-2014). This increasing trend was seen in adults (45-54 years, P < 0.001) and was predominant in males (77.6%). Hypertension (40.6%), hyperlipidemia (17.6%), and obesity (15%) were prevalent medical comorbidities with variable trends over the five years. Among substance use disorders, tobacco (50.9%), and alcohol (31.4%) were major comorbidities with a variable trend (P = 0.003 for each). There was a 71.4% increase in the inpatient mortality rate between 2010 (0.7%) and 2014 (1.2%). The mean length of stay was three days, and the total hospitalization charges have been increasing (P < 0.001), averaging $35,812 per hospital admission.

Conclusion

Chronic cannabis use or abuse worsens hospitalization outcomes in arrhythmic patients, and more clinical studies are needed to study the causal association between these conditions due to the rising mortality risk.

## Introduction

Arrhythmia describes an irregular heartbeat - the heart may beat too rapidly (tachycardia), too slowly (bradycardia), too early (premature contraction), or irregularly (flutter or fibrillation). Any interruption to the electrical impulses that cause the heart to contract can result in arrhythmia. Several factors can cause the heart to work incorrectly, including alcohol abuse, diabetes, substance abuse, excessive coffee consumption, congestive heart failure, hypertension, hyperthyroidism, mental stress, scarring of the heart due to a heart attack, and smoking. A healthy person will hardly ever suffer from long-term arrhythmia unless they have an external trigger, such as drug abuse or an electric shock. Most arrhythmias are non-fatal, but some can predispose the individual to stroke or cardiac arrest.

Currently, cannabis is used by a large proportion of the world population. It is produced on a large scale, and it is mainly used as an illicit substance by a large number of people across the world. It is estimated that 182.5 million people all over the world are using cannabis, which is about 3.8% of the total world population. Similarly, cannabis use is seen in the North American population, with an estimated annual prevalence of 11.6%, the majority within the age group of 15 to 64 years [1]. Since the majority of the people around the world, especially youth, use cannabis for recreational purposes, the trend of which is increasing globally, many people have led movements to decriminalize cannabis in different parts of the world. Recently, cannabis has been approved for use clinically for several medical conditions in various parts of the world. In the United States (US), a total of 25 states and the District of Columbia have allowed the use of cannabis for medical purposes [2].

Cannabis has arrhythmogenic properties [3-6]. Cannabis causes rapid development of tolerance to its effects with repeated exposures within a short period over one to two days. Chronic marijuana use causes a decrease in sympathetic activity and an increase in parasympathetic activity. These changes are reflected in our body by a decrease in heart rate (HR), increase in blood volume, absence of orthostatic hypotension, and decrease in circulatory response to exercise [6].

Several studies have demonstrated the arrhythmogenic properties of cannabis. A study on Norwegian apprehended drivers demonstrated that a higher mean pulse rate is seen in drivers who are tested positive for tetrahydrocannabinol (THC, an active ingredient of cannabis) as compared to drivers who are tested negative for THC. It also demonstrated that the rate of increase in HR was independent of the THC concentration in the blood [7]. A study on young people demonstrated that cannabis use causes atrial fibrillation in young people without any risk factors for atrial fibrillation in them [4,8]. In a systematic review of six case reports, Korantzopoulos et al. [4] hypothesized that cannabis use causes adrenergic stimulation and a decrease in the duration of the action potential. It causes changes in the electrophysiological properties of the myocardium, which favors automaticity and micro re-entry. Cannabis has a detrimental effect on coronary microcirculation and it causes atrial ischemia. Based on this, they concluded that cannabis smoking causes atrial fibrillation [4]. There are few case reports on cannabis use and ventricular tachycardia/fibrillation that may be due to excessive catecholamine release [9-11]. Some case reports demonstrated that excessive cannabis consumption causes a shortening of action potential and an increase in the vagal tone, which leads to Brugada-like ST-segment abnormalities [12-14].

The objective of this study is to study the trends of hospitalizations for arrhythmias with cannabis use disorder (CUD) in terms of demographic characteristics and inpatient outcomes, including the severity of illness, length of stay (LOS), and total charges during hospitalization.

## Materials and methods

Data source

We performed a retrospective analysis of the nationwide inpatient sample (NIS) data from January 2010 to December 2014 from the healthcare cost and utilization project (HCUP) [15]. The NIS provides discharge data from 4,400 hospitals across 45 states in the US, and when discharge weights are applied, the analysis result is a weighted estimate of nationally-representative total hospitalizations [15]. Diagnostic information in the NIS is detected using the international classification of diseases, ninth edition (ICD-9) codes and clinical classification software (CCS) codes [16].

Inclusion criteria and outcome variables

We included patients (age 15 to 54 years) with a primary discharge diagnosis of cardiac arrhythmia using the ICD-9 codes 427.0-427.2, 427.31, 427.32, 427.60, 427.61, 427.69, 427.81, 427.89, 427.9, 785.0, or 785.1) and a secondary discharge diagnosis of CUD using the ICD-9 codes 304.30, 304.31, 304.32, 305.20, 305.21, or 305.22 [17].

Demographic variables studied included age group (15-24, 25-34, 35-44, 45-54), gender (male or female), and race (Caucasian, African American, Hispanic, or other) [18]. The existing risk factors for cardiac arrhythmia were based on the past literature and identified using ICD-9 diagnosis codes and CCS codes.

We evaluated the differences in inpatient outcomes in the study population, and the outcome variables comprised the severity of illness (loss of functions measured as minor, moderate, and major) and in-hospital mortality (number of deaths during hospitalization) [18]. In the NIS, LOS is calculated as the total number of nights the patient was hospitalized for arrhythmia management and the total charges during hospitalization do not include professional fees and other non-covered charges [18].

Statistical analysis

We used the linear-by-linear association test for assessing the differences in demographics, comorbidities, and inpatient outcomes and the independent sample T-test for evaluating the changes seen in LOS and total charges over the study period from 2010 to 2014. A P-value less than 0.05 was used to determine the statistical significance and the analysis was conducted on the statistical package for the social sciences (SPSS) version 25 (IBM Corporation, Armonk, NY, US).

Ethical approval

Individual identifiers [18] were used to protect the patient's identity and other clinical information. The use of NIS under the HCUP does not require approval from the institutional review board, as the NIS is a publicly available de-identified database [15].

## Results

We analyzed 570,556 patients with a principal discharge diagnosis of arrhythmias from 2010 to 2014, from which 14,426 inpatients had comorbid CUD (2.53%). Arrhythmia inpatients with CUD were mostly males (77.6%) and adults (age 45 to 54 years, 43.4%). About half of them were Caucasians (51.2%) followed by African Americans (34.1%), Hispanics (9.8%), and other races or ethnicities (4.8%).

There was an increasing trend in arrhythmia hospitalizations with comorbid CUD from 2,536 (in 2010) to 3,325 (in 2014), representing a 31% increase over five years. There are variable trends in CUD with arrhythmia over the years from 2010 to 2014 in the age groups 15-24, 25-34, and 35-44 (P < 0.001). There is a statistically significant increasing trend seen in the age group 45-54 (P < 0.001). Arrhythmia was predominant in male cannabis users (77.6%), but there was a variable trend seen from 2010 to 2014. Differences between races showed statistically non-significant and variable trends (P = 0.474) among Caucasians (51.2%), African Americans (34.1%), Hispanics (9.8%), and other races or ethnicities (4.8%).

Hypertension (40.6%), hyperlipidemia (17.6%), and obesity (15%) were the most common medical comorbidities found in arrhythmia inpatients with CUD. There are variable trends in arrhythmia with CUD patients with comorbid diabetes, hypertension, obesity, elevated cholesterol, and lipids over the five-year study period. Among substance use disorders, tobacco (50.9%) and alcohol (31.4%) were significant comorbidities in these inpatients. Variable trends are seen with comorbid alcohol and tobacco use disorder in arrhythmia with CUD patients with statistical significance (P = 0.003), as shown in Table 1.

**Table 1:** Demographic trends of arrhythmia inpatients with cannabis use disorder

Variable	2010	2011	2012	2013	2014	Total	P value
Number of arrhythmia inpatients	122592	122749	116610	108050	100555	570556	-
Number of arrhythmia inpatients with cannabis use disorder	2536	2885	2875	2805	3325	14426	-
Prevalence	2.0	2.3	2.4	2.6	3.3	2.5	
Age at admission, in %							
15 – 24 years	12.6	16.0	12.0	14.6	12.9	13.6	<0.001
25 – 34 years	25.0	22.3	21.0	23.4	20.0	22.2	
35 – 44 years	20.7	20.7	22.8	18.0	21.5	20.8	
45 – 54 years	41.7	41.0	44.2	44.0	45.6	43.4	
Sex, in %							
Male	76.3	77.3	75.0	78.4	80.6	77.6	<0.001
Female	23.7	22.7	25.0	21.6	19.4	22.4	
Race, in %							
Caucasian	47.2	54.2	51.1	51.5	51.6	51.2	0.474
African American	38.3	31.9	33.2	31.8	35.6	34.1	
Hispanic	9.6	9.5	10.7	11.4	8.2	9.8	
Other	4.8	4.3	5.1	5.3	4.6	4.8	
Comorbid risk factors, in %							
Diabetes	9.8	10.0	9.2	10.7	12.2	10.5	0.001
Hypertension	34.8	42.8	40.2	39.2	44.7	40.6	<0.001
Obesity	9.1	16.4	17.6	14.4	16.5	15.0	<0.001
Elevated cholesterol and lipids	14.8	18.1	20.0	17.5	17.3	17.6	0.104
Alcohol use disorder	32.3	34.7	28.3	32.3	29.6	31.4	0.003
Tobacco use disorder	50.6	51.4	44.3	55.1	52.9	50.9	0.003

Majority of the inpatients with CUD were admitted for arrhythmia on a non-elective basis (95.8%). There are variable trends in arrhythmic inpatients with CUD developing minor, moderate, and major severity of illness over the years from 2010 to 2014 with statistical significance (P < 0.001). The mean number of chronic conditions is also variable with statistical significance (P < 0.001). Arrhythmia inpatients with CUD had a variable trend (P < 0.001) in inpatient mortality, but there is 71.4% increase in the inpatient mortality rate between 2010 (0.7%) and 2014 (1.2%). Trends in inpatient outcomes in arrhythmia hospitalizations with CUD are shown in Table 2. 

**Table 2:** Inpatient outcome trends of arrhythmia inpatients with cannabis use disorder SD: standard deviation

Variable	2010	2011	2012	2013	2014	Total	P value
Admission type, in %							
Non-elective	95.0	95.7	96.0	97.0	95.3	95.8	0.005
Elective	5.0	4.3	4.0	3.0	4.7	4.2	
Severity of illness, in %							
Minor	34.6	32.7	35.7	38.3	30.5	34.2	<0.001
Moderate	40.1	38.8	44.5	39.8	42.7	41.2	
Major	25.4	28.6	19.8	21.9	26.8	24.6	
Mean number of chronic conditions (SD)	5.8 (2.57)	6.2 (2.77)	6.3 (2.82)	6.3 (2.84)	6.7 (2.91)	6.3 (2.81)	<0.001
In-hospital mortality, in %							
Inpatient deaths	0.7	0.7	1.4	0.2	1.2	0.9	<0.001
Other outcomes							
Mean length of stay (SD), in days	2.9 (2.89)	2.8 (3.07)	2.8 (3.10)	3.2 (6.03)	3.2 (3.27)	3.0 (3.85)	<0.001
Mean total charges (SD), in $	33561.94	31614.48	31192.38	37891.46	43470.28	35812.28	<0.001

**Figure 1:**
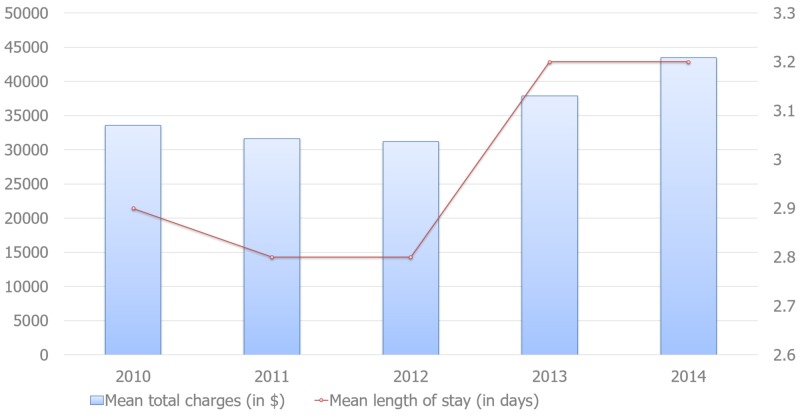
Trends in inpatient mean length of stay and total charges, 2010-2014

The mean LOS was three days, and it remains more or less around three days over the study period with statistical significance (P < 0.001), whereas total hospitalization charges have been increasing significantly (P < 0.001), averaging $35,812 per hospital admission, as shown in Figure 1.

## Discussion

This study describes the trend examination of arrhythmia hospitalizations and comorbid CUD using the US population-based inpatient data during the post-legalization period (2010 to 2014). Our study findings are supporting existing literature about cannabis abuse and dependence and its impact on vascular function and arrhythmia [3-6,19-20].

Cannabis has arrhythmogenic properties. Cannabis smoking causes reflex tachycardia due to cannabis-induced vasodilation [3-4]. It causes a 20% to 100% increase in HR. It also causes a slight increase in supine blood pressure. This increase in HR lasts for about two to three hours. Cannabis causes postural hypotension associated with dizziness and fainting when consumed in higher doses [5-6].

Our study showed a significant increase in arrhythmia hospitalizations with comorbid CUD by 31% during the post-legalization period that brings an important public health concern. CUD may be an associated risk factor for hospitalization due to its arrhythmogenic properties. These patients have various comorbid conditions like diabetes, hypertension, obesity, elevated cholesterol and lipids, and alcohol and tobacco abuse. These risk factors also play a significant role in the development of arrhythmia in patients with cannabis abuse or dependence [4,7-8]. These comorbid risk factors may have synergistic effects with cannabis and potentially increase the chances of arrhythmia hospitalization.

We found that a higher proportion of arrhythmia hospitalizations with CUD were in adults aged 45 to 54 years, which may be due to an increase in comorbid conditions at an older age [21]. There are few case reports on chronic cannabis use and ventricular tachycardia/fibrillation that may be due to excessive catecholamine release [10-11]. It is speculated that excessive catecholamine release in cannabis users could be responsible for the arrhythmia [11]. In our study, 52.9% arrhythmia inpatients with CUD have comorbid tobacco abuse or dependence, which indicates an important problem of tobacco in cannabis users that may increase the chances of arrhythmia hospitalizations [22].

The study highlights the increase in arrhythmia hospitalizations, total inpatient charges, and mortality in patients with comorbid CUD. This is mainly reflected by several findings in our study, including increase in the mean length of stay (three days), average hospitalization total charges of $35,812, and 71.4% increase in in-hospital mortality during the management of arrhythmia [20,23]. The increase in hospitalization costs is due to an increase in the overall severity of illness by 5.5% [20] and a number of chronic conditions by 15.52% [21,24-25].

There are some limitations to our study. As per our knowledge, this is one of the pioneer studies; we did not have enough literature to support our results adequately. Our study nevertheless utilizes the NIS dataset and lays the basis for these results, which will have substantial consequences for future studies. The second limitation was that the readmission status for the participants could not be looked upon, and this is due to the nature of the database. However, since this database is a robust population-based register with a high generalisability, it outweighs the significance of these results in comparison to limitations for this study. This research nevertheless possesses some strengths. First, in the form of generalization of outcomes, our research shows strong internal validity. Secondly, this is the first study that evaluates the patterns and effects of CUD on arrhythmia hospitalization and its impact on inpatient outcomes. The study findings indicate that healthcare expenses are increasing inadvertently, primarily due to the use of cannabis and to avoidable risk factors. The ultimate strength of the study is how the study eliminates a reporting bias by using the NIS dataset, and its distinctive feature is that information is coded separately from the physician.

## Conclusions

There is a decreasing trend in the number of arrhythmia cases in inpatient US hospitalizations, but there is an increase in the number of inpatient hospitalizations with arrhythmia having CUD as per our study. A statistically significant rising trend of arrhythmia hospitalization with CUD is seen in adults (45 to 54 years), males, and Caucasians. Cardiometabolic comorbidities, including diabetes, hypertension, and obesity, had an increasing linear trend in these inpatients. Hence, there was a spike seen in the severity of illness and the number of chronic conditions, which may have indirectly attributed to the increasing hospitalization costs for arrhythmia management in cannabis users. Despite all these measures, in-hospital mortality rose by 71.4% during the post-legalization period. The outcomes in arrhythmic patients with CUD are presumably worse due to chronic cannabis abuse. More clinical studies are required to demonstrate a causal association between episodic cannabis use and arrhythmia. Since medical cannabis laws are passed in the US, large-scale epidemiological studies are also needed to assess the potential risks in cannabis users due to an increase in the use of cannabis for therapeutic purposes.
